# Proportions of *Pseudomonas aeruginosa* and Antimicrobial-Resistant *P aeruginosa* Among Patients With Surgical Site Infections in China: A Systematic Review and Meta-analysis

**DOI:** 10.1093/ofid/ofad647

**Published:** 2023-12-18

**Authors:** Yuhui Yang, Li Zhang, Jian Wang, Zongyue Chen, Liang Tong, Zhenkun Wang, Gaoming Li, Yu Luo

**Affiliations:** School of Nursing, Army Medical University, Chongqing, China; Disease Surveillance Division, Center for Disease Control and Prevention of Central Theater Command, Beijing, China; Department of Prevention and Control of Infectious Diseases, Center for Disease Control and Prevention of Central Theater Command, Beijing, China; School of Nursing, Army Medical University, Chongqing, China; Disease Surveillance Division, Center for Disease Control and Prevention of Central Theater Command, Beijing, China; Disease Surveillance Division, Center for Disease Control and Prevention of Central Theater Command, Beijing, China; Disease Surveillance Division, Center for Disease Control and Prevention of Central Theater Command, Beijing, China; School of Nursing, Army Medical University, Chongqing, China

**Keywords:** antimicrobial resistance, meta-analysis, *Pseudomonas aeruginosa*, surgical site infections

## Abstract

**Background:**

*Pseudomonas aeruginosa* is one of the most common pathogens in surgical site infections (SSIs). However, comprehensive epidemiological and antibiotic resistance details for *P aeruginosa* in Chinese SSIs are lacking. We evaluated the proportions and antimicrobial resistance of *P aeruginosa* among patients with SSIs in China.

**Methods:**

Relevant papers from January 2010 to August 2022 were searched in databases including PubMed, Embase, Web of Science, China Biomedical Literature Database, China National Knowledge Infrastructure, Wanfang, and Weipu. A meta-analysis was performed to analyze the proportions and 95% confidence interval (CIs) of *P aeruginosa* among patients with SSIs. Meta-regression analysis was used to investigate the proportion difference among different subgroups and antimicrobial resistance.

**Results:**

A total of 72 studies met inclusion criteria, involving 33 050 isolated strains. The overall proportion of *P aeruginosa* among patients with SSIs was 16.0% (95% CI, 13.9%–18.2%). Subgroup analysis showed higher proportions in orthopedic (18.3% [95% CI, 15.6%–21.0%]) and abdominal surgery (17.3% [95% CI, 9.9%–26.2%]). The proportion in the central region (18.6% [95% CI, 15.3%–22.1%]) was slightly higher than that in other regions. Antibiotic resistance rates significantly increased after 2015: cefoperazone (36.2%), ceftriaxone (38.9%), levofloxacin (20.5%), and aztreonam (24.0%). Notably, *P aeruginosa* resistance to ampicillin and cefazolin exceeded 90.0%.

**Conclusions:**

The proportion of *P aeruginosa* infection among patients with SSIs was higher than the data reported by the Chinese Antimicrobial Resistance Surveillance System, indicating rising antimicrobial resistance. The existing antimicrobial drug management plan should be strengthened to prevent a hospital epidemic of drug-resistant *P aeruginosa* strains.

Surgical site infections (SSIs) are the most common and challenging nosocomial infections in surgical patients worldwide. Approximately 230 million surgeries are performed each year worldwide, of which approximately 31% result in patients with varying degrees of SSIs; importantly, one-third of postoperative deaths are related to SSIs [1–4]. The incidence of SSIs varies among countries with different economic levels. In Europe, the SSI rate is 2%–5%; in Africa, it is as high as 51.1% [5]. It is estimated that in the United States, 2.5% of surgical patients will have varying degrees of SSIs each year. Compared with that for patients without infections, the average length of hospital stay for patients with SSIs is 7–11 days longer and the risk of death is 2–11 times higher than those for patients without infection. The annual total cost associated with SSIs exceeds $10 billion [6–8]. In short, SSIs not only bring a heavy financial burden to patients but are also life-threatening.

Although the pathogenic microorganisms that cause infection are diverse and the epidemiology of SSIs is also different among different environments and different countries, it has been reported that the proportions of gram-negative bacilli increases over time and that *Pseudomonas aeruginosa* is one of the most common pathogens [9]. In a hospital in Egypt, 70 strains of *P aeruginosa* were detected in 200 SSI samples (the detection rate was 35.0%) [10]. In a tertiary referral hospital in Amman, Jordan, a large number of gram-negative bacteria were detected in SSI samples, of which *P aeruginosa* was one of the main pathogenic bacteria; the detection rate was 14.8% [11].

The increase in antibiotic resistance of pathogens has become a major public health problem. Among them, multidrug-resistant (MDR) *P aeruginosa* has become increasingly serious in nosocomial infections and is closely related to the increase in mortality and length of hospital stay [12]. In 2017, the World Health Organization (WHO) published the first list of antibiotic-resistant “key pathogens.” There are 3 extremely important pathogens, including carbapenem-resistant *P aeruginosa* [13]. The report of the National Notifiable Disease Surveillance System showed that the antimicrobial resistance rates for *P aeruginosa* to imipenem, quinolones, and third-generation cephalosporins were 15%, 9%, and 20%, respectively [8]. According to WHO data, the antimicrobial resistance rates of *P aeruginosa* to fluoroquinolones, ceftazidime, and aminoglycosides in 2020 were 46.4%, 41.0%, and 37.1%, respectively [14, 15]. The antimicrobial resistance mechanisms of *P aeruginosa* are complex and diverse, and some strains can develop resistance to multiple antibiotics at the same time, posing a great challenge to the selection of appropriate antibiotics. Previous studies have found that the most suitable antibiotics for MDR *P aeruginosa* are carbapenems. However, *P aeruginosa* produces large amounts of metallo-β-lactamases, leading to resistance to β-lactams, including carbapenems, greatly limiting the choice of the correct treatment plan [16].

In China, data from the National Nosocomial Infection Surveillance System showed that SSIs accounted for 10.4% of all nosocomial infections and that the rate of detection of *P aeruginosa* in SSIs was 8.9% [17]. In 2022, data from the Chinese Antimicrobial Resistance Surveillance System indicated that the antimicrobial resistance rates of *P aeruginosa* to imipenem and meropenem steadily declined in the past 5 years, fluctuating between 18.9% and 30.7%; the antimicrobial resistance rate to polymyxin B was low, fluctuating between 0.5% and 1.2%, consistent with the data reported in 2020; and the antimicrobial resistance rates to gentamycin, ciprofloxacin, ceftazidime, cefepime, and piperacillin were below 20.0%. Chinese clinicians have gradually realized that the antimicrobial resistance of *P aeruginosa* is becoming increasingly serious, especially the increasing number of *P aeruginosa* strains capable of producing metallo-β-lactamases, leading to the ineffectiveness of multiple antibiotics in the treatment of SSIs and increasing the physical and economic burden on patients [18].

Understanding the epidemiological characteristics and antibiotic resistance of *P aeruginosa* that cause SSIs is critical for clinicians to develop prevention and treatment measures for SSIs. Although previous studies have described the epidemiological characteristics and antimicrobial resistance of *P aeruginosa* in nosocomial infections, substantial uncertainty remains in the proportions of *P aeruginosa* among SSIs. Therefore, the purpose of this systematic review was to summarize and assess the proportions and antimicrobial resistance rate of *P aeruginosa* among SSIs to provide further guidance for the prevention of SSIs and to promote the best empirical antimicrobial treatment.

## METHODS

The study was conducted in accordance with the Preferred Reporting Items for Systematic Reviews and Meta-Analyses (PRISMA) statement [19] and registered on the PROSPERO database (CRD42022363001).

### Search Strategy

The study used free words combined with subject terms to comprehensively search the PubMed, Embase, Web of Science, China Biomedical Literature Database, China National Knowledge Infrastructure, Wanfang, and Weipu databases (Supplementary Table 1). The focus of this study was to analyze the epidemiological and antimicrobial resistance characteristics of *P aeruginosa* in the past decade. Therefore, the search time was limited to January 2010 to August 2022. The search terms included surgery, postoperative, surgical wound infection, site infection, *P aeruginosa*, and China. The retrieved papers were managed using EndNote (version 20), and duplicates were eliminated. Relevant conference papers were manually searched in the journal database of the Army Medical University Library, and all references included in the study were reviewed.

### Inclusion and Exclusion Criteria

The literature included in the meta-analysis met the following criteria: (1) patients were clinically diagnosed with SSIs; (2) the sample collection began in 2010; (3) the study subjects were Chinese; and (4) there were sufficient data to calculate the proportions or antimicrobial resistance of *P aeruginosa*. The following exclusion criteria were applied: (1) abstracts, reviews, or communication papers; (2) studies with a small number of detected bacterial strains (requiring at least 197 isolated bacterial strains [20] to estimate the expected proportion of *P aeruginosa* at 8.9% [17] and an error rate of <4%); (3) a lack of sufficient information, including incomplete or unavailable research data; and (4) research based on data from the National Nosocomial Infection Surveillance System.

### Data Extraction and Bias Risk Assessment

Data were extracted independently by 2 researchers (Y. Y. and G. L.) using a unified data table. The main data extracted included the first author, publication year, study area, study design, hospital level, and data that were used to calculate the proportions of *P aeruginosa* and drug-resistant *P aeruginosa*. For conflicts regarding data extraction, a consensus was reached through consultation with a third party. For duplicated publications, only data with the highest quality and highest number of detected bacterial strains or the most complete information were extracted.

The quality of the included literature was evaluated using predetermined criteria extracted and modified from a previous case series scale consisting of 8 items [21]. By answering low, high, or unclear to questions, bias can be identified in selected literature. The total score ranges from 0 to 8 points, and the higher the score is, the higher the quality. In this study, R version 4.1.3 software was used to summarize the risk of bias. A score <4 points was defined as high risk, and a score ≥4 points was defined as low risk.

### Statistical Analysis

All statistical analyses were performed using R version 4.1.3 software. Statistical tests were all 2-tailed. Unless stated, *P* < .05 was considered statistically significant. The proportions of *P aeruginosa* and antimicrobial-resistant *P aeruginosa* isolates among SSI patients in each study were calculated using the following formula:


$$\mathrm{Proportions}\;\mathrm{of}\;Pseudomonasaeruginosa=\frac{\mathrm{Number}\;\mathrm{of}\;Pseudomonasaeruginosa\;\mathrm{isolates}\;}{\mathrm{Number}\;\mathrm{of}\;\mathrm{all}\;\mathrm{the}\;\mathrm{detected}\;\mathrm{isolates}}\times 100\text{\%}$$

Proportionsofantimicrobial-resistantPseudomonasaeruginosa=NumberofdetectedPseudomonasaeruginosaisolatesresistanttoagivenantibioticNumberofPseudomonasaeruginosaisolatesdetected×100%

The proportion in each study and its 95% confidence interval (CI) were calculated using Freeman-Tukey double arcsine transformation [22].

A DerSimonian-Laird random effects model was used to estimate the combined proportions of the included literature and its corresponding 95% CI [23]. The Cochrane *Q* test was used to analyze the heterogeneity among the studies. The *Q* statistic approximately follows the *χ^2^* distribution with *k* – 1 (*k* is the number of studies). When *P* is <.10, it can be considered that there is heterogeneity between studies. The magnitude of heterogeneity was quantitatively evaluated based on the Higgins *I*^2^ value, which ranges from 0% to 100%, and significant heterogeneity is generally considered to exist for *I*^2^ values >50% [24]. A funnel plot was used to analyze whether there was publication bias in the included literature, and Egger linear regression was used to test the asymmetry of the funnel plot [25].

The differences in the proportions of *P aeruginosa* and the antimicrobial resistance rate for *P aeruginosa* were explored through subgroup analysis and univariate meta-regression. In the meta-regression analysis, the dependent variable was the proportion of *P aeruginosa* or the antibiotic resistance data for *P aeruginosa* isolates. The independent variables were surgical type (dummy variable: Orthopedic), region (dummy variable: Eastern region), hospital level (dummy variable: Tertiary), risk of bias (dummy variable: High), study design (dummy variable: Retrospective), sample size (dummy variable: <500 isolates), and study time (dummy variable: 2015 or after). In the meta-regression analysis, the restricted maximum likelihood method was used to estimate the variance between studies, and the proportion of variance explained by any meta-regression model was estimated using the *R*^2^ statistic [26, 27].

## RESULTS

### Literature Search

**Figure 1.**
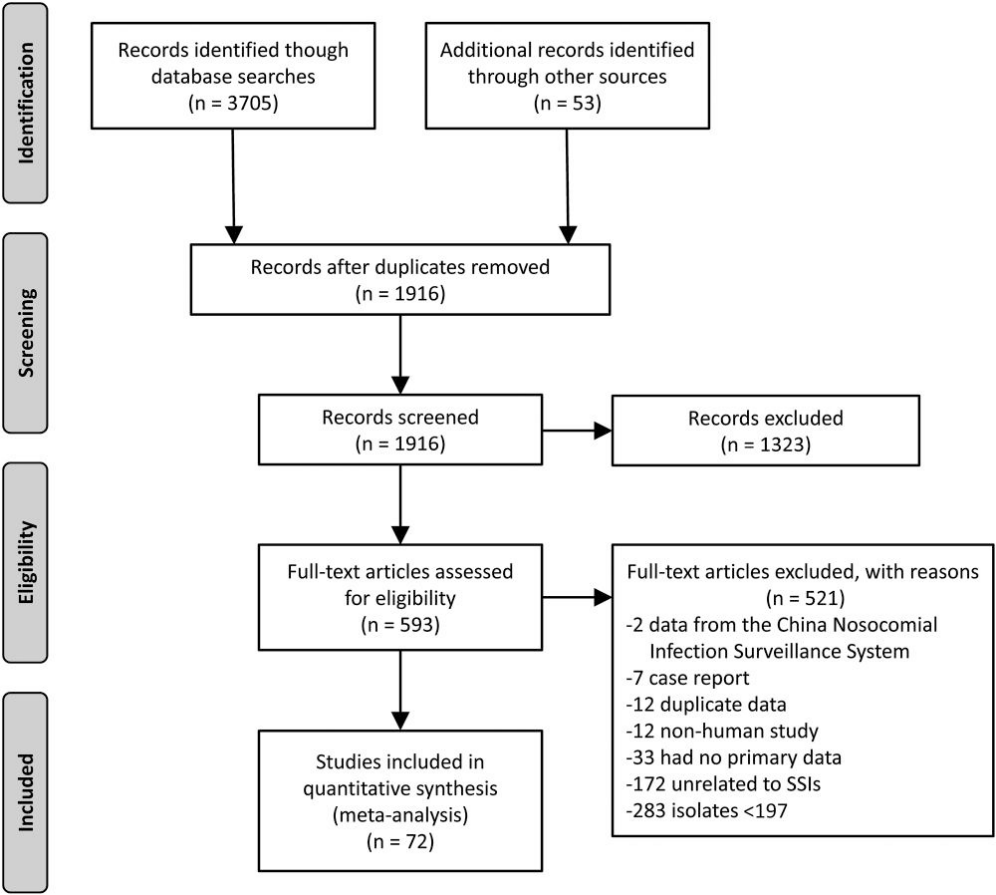
Literature inclusion and exclusion process.

The initial search obtained 3758 relevant studies. After excluding duplicates, we reviewed the titles and abstracts of 1916 papers (preliminary screening) and excluded 1323 studies. After further reading the abstracts and full texts, we excluded 521 papers. Among them, there were 283 studies with a total number of isolated bacterial strains <197, 172 studies with nonsurgical wound infections, 33 studies without valid data, 12 studies with nonhuman subjects, 12 duplicated publications, 7 case reports, and 2 studies based on the National Nosocomial Infection Surveillance System. Ultimately, 72 studies that met the inclusion criteria were included in the analysis (Figure 1).

### Study Characteristics and Bias Risk Assessment

**Figure 2.**
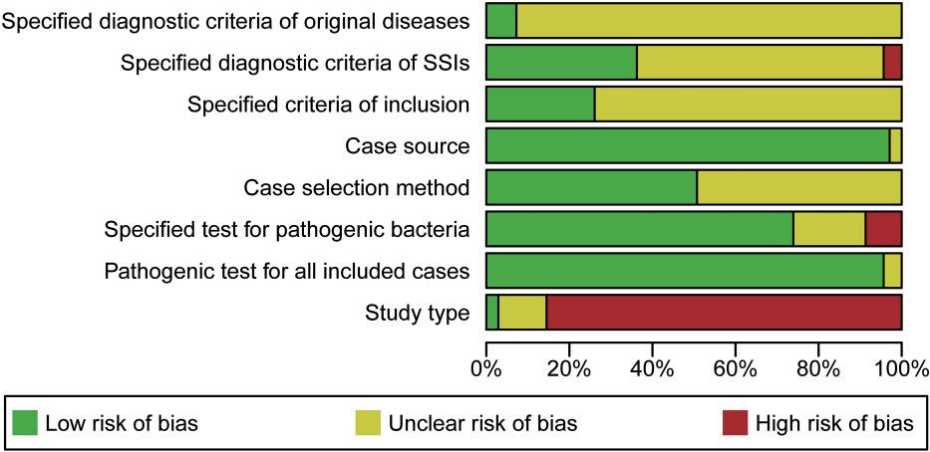
Results of the risk of bias assessment. Abbreviation: SSI, surgical site infection.

**Figure 3.**
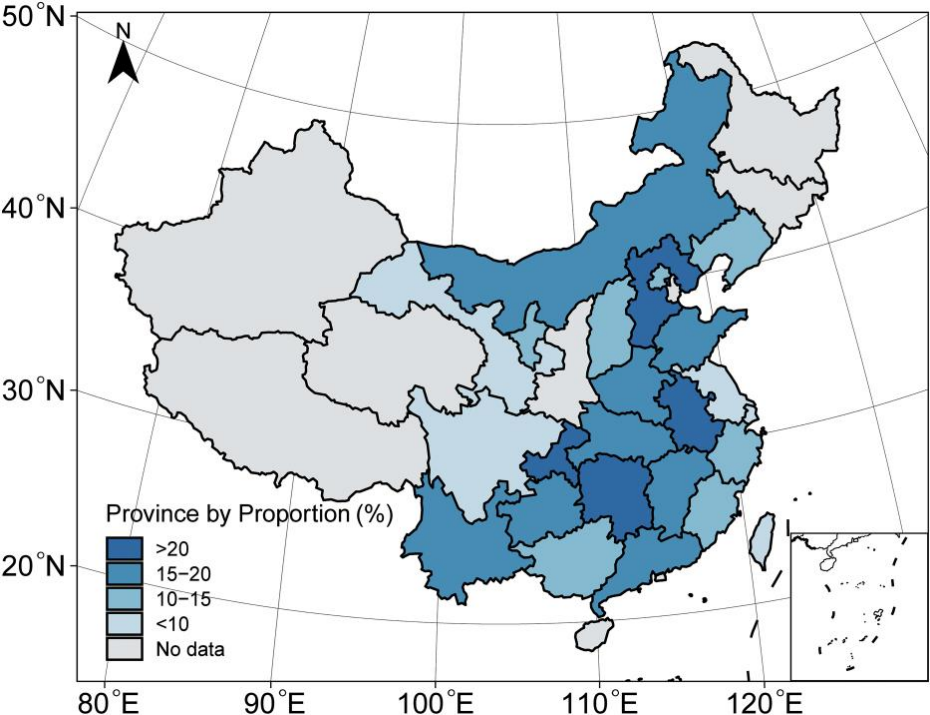
Geographic distribution of *Pseudomonas aeruginosa* proportions among patients with surgical site infections in China.

The characteristics of the 72 included studies are shown in Table 1. Among them, 69 studies reported the proportion of *P aeruginosa* among SSIs. The number of bacterial strains isolated per study ranged from 201 to 2162, with a total of 33 050 strains isolated. Another 3 studies reported antimicrobial resistance data for *P aeruginosa*. The results of the risk of bias assessment are shown in Figure 2, and the details are shown in Supplementary Table 2. The highest score for a single study was 7, and the lowest score was 1. There were 48 high-quality studies (≥4). All studies were conducted between 2010 and 2023, and 24 of the 34 provinces in China were represented (Figure 3). Among them, 28 studies were from the eastern coastal region, 33 studies were from the central region, and 11 studies were from the western region. Most studies were retrospective (62 of 72).

**Table 1.** General Characteristics of the Studies Included in the Meta-analysis

Study ID	Year(s)	Province	Study Type	Surgery Type^a^	Hospital Level	No. of SSI Patients	Isolates	*Pseudomonas aeruginosa*	Risk of Bias
Chen 2012 [28]	2010	Taiwan	Retrospective	Abdominal	Tertiary	117	221	18	Low
Gao 2013 [29]	2010–2012	Hebei	Retrospective	Orthopedic	Tertiary	999	1056	245	Low
Han 2013 [30]	2010–2011	Shanxi	Retrospective	Orthopedic	Tertiary	Unclear	619	73	High
You 2013 [31]	2010–2012	Fujian	Retrospective	Multiple	Tertiary	Unclear	588	50	High
Zhao 2013 [32]	2010–2012	Guangxi	Retrospective	Multiple	Tertiary	4459	1442	185	High
Feng 2014 [33]	2012–2013	Henan	Retrospective	Multiple	Nontertiary	299	258	22	High
Gao 2014 [34]	2012	Hebei	Monitoring	Orthopedic	Tertiary	223	247	63	Low
Guo 2014 [35]	2010–2013	Tianjin	Retrospective	Multiple	Tertiary	Unclear	Unclear	186	High
Huang 2014 [36]	2012–2013	Shandong	Retrospective	Orthopedic	Tertiary	127	356	89	High
Liu 2014 [37]	2014	Hunan	Retrospective	Orthopedic	Tertiary	243	243	59	High
Mo 2014 [38]	2012–2013	Henan	Prospective	Orthopedic	Tertiary	Unclear	201	32	Low
Zheng 2014 [39]	2012–2013	Henan	Retrospective	Orthopedic	Tertiary	651	407	52	High
Chen 2015 [40]	2010–2013	Hubei	Retrospective	Orthopedic	Tertiary	200	210	33	High
Lou 2015 [41]	2011–2014	Henan	Retrospective	Multiple	Tertiary	1962	988	112	Low
Ran 2015 [42]	2013–2014	Chongqing	Prospective	Multiple	Nontertiary	241	270	57	Low
Su 2015 [43]	2013–2014	Guangdong	Monitoring	Orthopedic	Nontertiary	3317	603	66	Low
Wang 2015 [44]	2014	Shandong	Retrospective	Orthopedic	Tertiary	360	404	76	High
Xie 2015 [45]	2011–2013	Henan	Monitoring	Abdominal	Tertiary	248	234	26	Low
Yang 2015 [46]	2011–2013	Hubei	Retrospective	Multiple	Nontertiary	377	407	88	Low
Yao 2015 [47]	2013–2014	Zhejiang	Monitoring	Multiple	Nontertiary	210	204	22	Low
Zhong 2015 [48]	2011–2014	Guangdong	Retrospective	Orthopedic	Nontertiary	300	920	300	Low
Dong 2016 [49]	2014–2015	Hebei	Retrospective	Orthopedic	Tertiary	2106	2106	502	Low
Hu 2016 [50]	2011–2015	Zhejiang	Retrospective	Abdominal	Nontertiary	263	227	35	Low
Peng 2016 [51]	2011–2014	Hubei	Retrospective	Abdominal	Tertiary	188	237	41	Low
Wang 2016 [52]	2010–2015	Inner Mongolia	Retrospective	Abdominal	Tertiary	186	203	48	High
Wang 2016 [53]	2010–2014	Liaoning	Retrospective	Orthopedic	Tertiary	915	915	258	High
Yang 2016 [54]	2013–2015	Shandong	Retrospective	Multiple	Tertiary	989	887	78	Low
Yu 2016 [55]	2012–2014	Henan	Retrospective	Abdominal	Tertiary	1022	1020	455	High
Chen 2017 [56]	2012–2014	Henan	Retrospective	Abdominal	Tertiary	218	218	48	Low
Jin 2017 [57]	2014–2016	Zhejiang	Retrospective	Orthopedic	Nontertiary	232	296	45	Low
Li 2017 [58]	2011–2013	Hebei	Retrospective	Orthopedic	Tertiary	247	278	62	Low
Liu 2017 [59]	2015	Jiangsu	Retrospective	Multiple	Tertiary	307	204	12	Low
Liu 2017 [60]	2012–2017	Zhejiang	Retrospective	Orthopedic	Nontertiary	70	327	61	Low
Luo 2017 [61]	2016	Henan	Retrospective	Craniocerebral	Nontertiary	66	210	32	High
Wan 2017 [62]	2014–2016	Henan	Retrospective	Orthopedic	Nontertiary	372	370	66	Low
Wu 2017 [63]	2011–2015	Zhejiang	Retrospective	Craniocerebral	Tertiary	23 259	419	17	Low
Wu 2017 [64]	2012–2015	Zhejiang	Retrospective	Orthopedic	Tertiary	397	397	34	Low
Ren 2018 [65]	2016–2017	Inner Mongolia	Retrospective	Multiple	Tertiary	450	286	43	High
Sun 2018 [66]	2015–2017	Jiangxi	Retrospective	Orthopedic	Tertiary	216	270	46	High
Xie 2018 [67]	2012–2016	Yunnan	Retrospective	Orthopedic	Tertiary	295	345	53	High
Yang 2018 [68]	2012–2017	Gansu	Retrospective	Multiple	Nontertiary	246	278	14	Low
Zhou 2018 [69]	2017–2018	Zhejiang	Retrospective	Orthopedic	Tertiary	106	251	42	High
Zhou 2018 [70]	2012–2017	Shandong	Retrospective	Multiple	Nontertiary	448	548	49	Low
Gao 2019 [71]	2012–2016	Shandong	Monitoring	Multiple	Nontertiary	654	247	39	Low
Hu 2019 [72]	2015–2018	Jiangxi	Retrospective	Orthopedic	Nontertiary	36	206	18	Low
Liang 2019 [73]	2015–2017	Henan	Retrospective	Orthopedic	Tertiary	280	324	64	High
Wang 2019 [74]	2015–2018	Anhui	Retrospective	Orthopedic	Tertiary	224	246	51	Low
Yu 2019 [75]	2017–2018	Jiangxi	Retrospective	Orthopedic	Nontertiary	72	382	136	Low
Chai 2020 [76]	201–2018	Henan	Retrospective	Spinal	Nontertiary	263	342	27	High
Gong 2020 [77]	2015–2018	Sichuan	Retrospective	Orthopedic	Tertiary	1020	1020	89	Low
Huang 2020 [78]	2018–2019	Shanghai	Retrospective	Abdominal	Tertiary	Unclear	450	36	Low
Lin 2020 [79]	2016–2019	Guangdong	Monitoring	Orthopedic	Nontertiary	409	228	30	Low
Pan 2020 [80]	2014–2018	Sichuan	Retrospective	Multiple	Tertiary	Unclear	Unclear	714	High
Wang 2020 [81]	2014–2018	Henan	Retrospective	Abdominal	Nontertiary	324	360	27	Low
Zhao 2020 [82]	2016–2018	Henan	Retrospective	Abdominal	Tertiary	226	834	284	Low
Dong 2021 [83]	2017–2020	Henan	Retrospective	Multiple	Tertiary	155	Unclear	155	High
Wang 2021 [84]	2015–2019	Henan	Retrospective	Orthopedic	Nontertiary	562	587	176	Low
Wang 2021 [85]	2020	Liaoning	Monitoring	Multiple	Tertiary	Unclear	811	89	Low
Xu 2021 [86]	2019	Gansu	Retrospective	Orthopedic	Tertiary	430	252	40	High
Yang 2021 [87]	2017–2019	Henan	Retrospective	Orthopedic	Tertiary	76	258	29	Low
Ye 2021 [88]	2018–2019	Fujian	Retrospective	Orthopedic	Tertiary	296	296	74	High
Yu 2021 [89]	2015–2018	Guangdong	Retrospective	Orthopedic	Tertiary	516	516	86	Low
Zheng 2021 [90]	2020	Fujian	Retrospective	Orthopedic	Tertiary	890	647	44	Low
Chen 2022 [91]	2018–2019	Henan	Retrospective	Orthopedic	Tertiary	265	290	60	Low
Li 2022 [92]	2019–2021	Henan	Retrospective	Orthopedic	Tertiary	95	463	167	Low
Ma 2022 [93]	2017–2018	Ningxia	Retrospective	Multiple	Tertiary	288	584	66	Low
Sun 2022 [94]	2011–2020	Beijing	Monitoring	Abdominal	Tertiary	1280	2162	228	Low
Wang 2022 [95]	2018–2021	Henan	Retrospective	Orthopedic	Tertiary	370	225	16	Low
Xie 2022 [96]	2012–2020	Guizhou	Retrospective	Multiple	Tertiary	580	449	78	Low
Cao 2023 [97]	2016–2021	Shandong	Retrospective	Orthopedic	Tertiary	203	242	82	Low
Han 2023 [98]	2018–2019	Liaoning	Retrospective	Orthopedic	Tertiary	212	243	18	Low
Zhang 2023 [99]	2021–2023	Henan	Retrospective	Others	Tertiary	136	216	32	Low

Abbreviation: SSI, surgical site infection.

^a^In this column, “multiple” refers to the different types of surgeries involved in the research and cannot be classified into a specific type of surgery.

### Proportions of *Pseudomonas aeruginosa*

**Figure 4.**
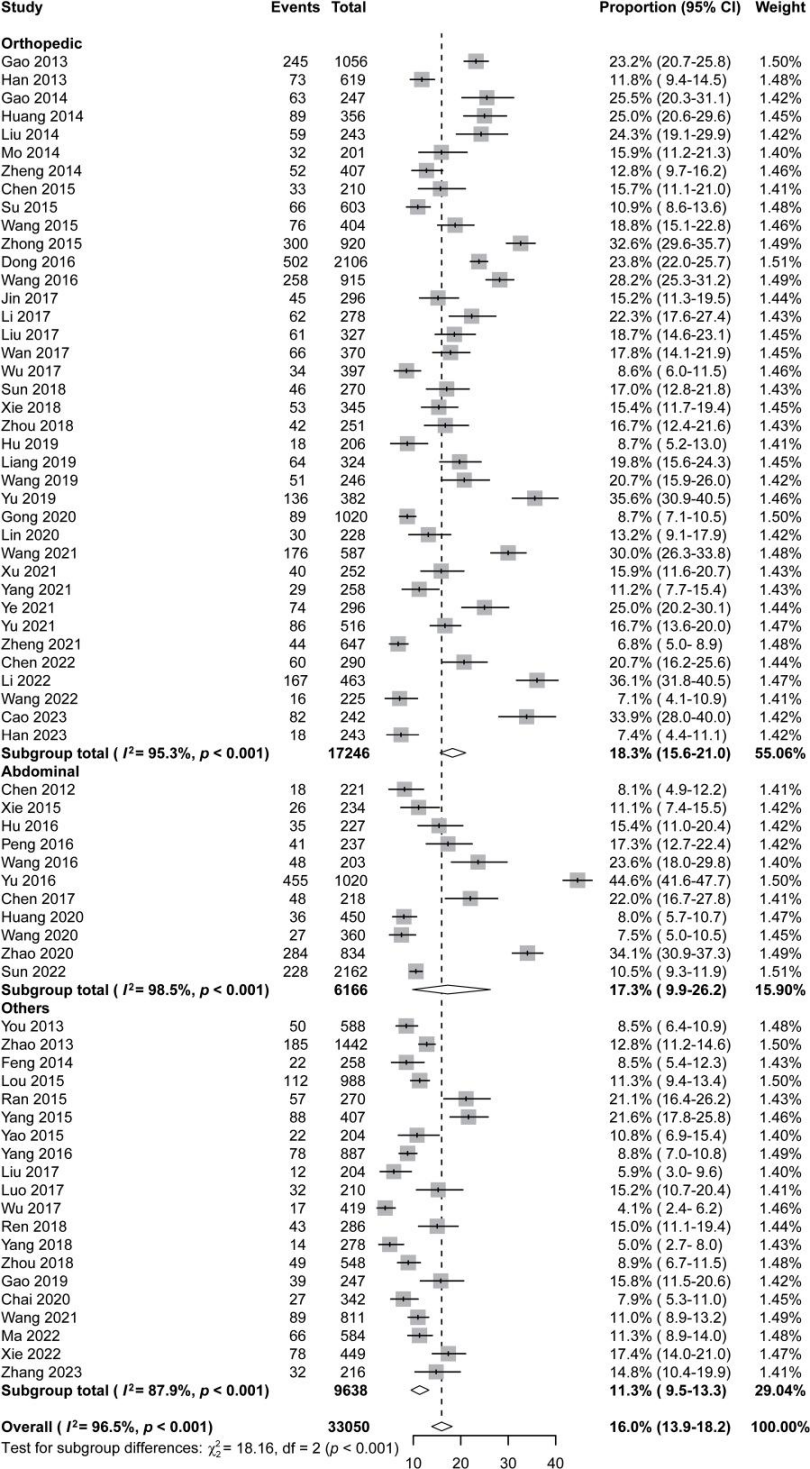
The proportions of *Pseudomonas aeruginosa* among patients with surgical site infections in different surgical subgroups. Abbreviation: CI, confidence interval.

The proportions of *P aeruginosa* included in the studies ranged from 4.1% (95% CI, 2.4%–6.2%) to 44.6% (95% CI, 41.6%–47.7%) (Figure 4), indicating significant heterogeneity among the studies. The Higgins *I*^2^ value was 96.5 (Q test *P* < .001). The overall proportion of *P aeruginosa* was 16.0% (95% CI, 13.9%–18.2%; Table 2); the proportion of *P aeruginosa* infection was 18.3% (95% CI, 15.6%–21.0%) in orthopedic surgery, 17.3% (95% CI, 9.9%–26.2%) in abdominal surgery, and 11.3% (95% CI, 9.5%–13.3%) in other types of surgery; the proportion was 13.8% (95% CI, 10.9%–16.9%) in the eastern region, 18.6% (95% CI, 15.3%–22.1%) in the central region, and 14.0% (95% CI, 11.1%–17.2%) in the western region. The proportions of *P aeruginosa* exceeded 20% in 4 provinces: 24.3% (95% CI, 19.1%–29.9%) in Hunan Province, 23.6% (95% CI, 22.2%–25.0%) in Hebei Province, 21.1% (95% CI, 16.5%–26.2%) in Chongqing City, and 20.8% (95% CI, 15.9%–26.1%) in Anhui Province. Univariate random effects meta-regression analysis indicated that the type of surgery (*R*^2^ = 12.84) and region (*R*^2^ = 5.90) might be potential sources of heterogeneity and that there were no significant differences in hospital level (*R*^2^ < 0.01), risk of bias (*R*^2^ = 0.25), study design (*R*^2^ < 0.01), sample size (*R*^2^ < 0.01), and study time (*R*^2^ < 0.01). The results of the Egger test indicated that there was no evidence of publication bias regarding the total proportion of *P aeruginosa* (*t* = –0.876, *P* = .384; Supplementary Figure 1).

**Table 2.** Proportions of *Pseudomonas aeruginosa* Infection Among Different Subgroups of Patients With Surgical Site Infections

Group	Proportions of *Pseudomonas aeruginosa*					Univariate Meta-regression		
	Studies	Isolates	*Pseudomonas aeruginosa*	Estimate, %	*I* ^2^	Coefficient (95% CI)	*P* Value	*R* ^2^
Surgery type								
Orthopedic	38	17 246	3437	18.3 (15.6–21.0)	95.3	Ref	Ref	12.84
Abdominal	11	6166	1246	17.3 (9.9–26.2)	98.5	−0.012 (−.083 to .060)	.744	…
Other^a^	20	9638	1112	11.3 (9.5–13.3)	87.9	−0.097 (−.155 to −.040)	.001	…
Region								
Eastern	27	13 609	1978	13.8 (10.9–16.9)	96.1	Ref	Ref	5.90
Central	32	14 312	3144	18.6 (15.3–22.1)	96.4	0.066 (.009 to .122)	.023	…
Western	10	5129	673	14.0 (11.1–17.2)	89.1	0.005 (−.075 to .085)	.902	…
Hospital level								
Tertiary	49	25 780	4485	16.2 (13.8–18.9)	96.8	Ref	Ref	<0.01
Nontertiary	20	7270	1310	15.3 (11.5–19.6)	95.8	−0.013 (−.072 to .047)	.676	…
Risk of bias								
High	21	9241	1821	17.6 (13.5–22.2)	96.6	Ref	Ref	0.25
Low	48	23 809	3974	15.3 (12.9–17.8)	96.4	−0.032 (−.090 to .026)	.280	…
Study design								
Retrospective	59	27 843	5143	16.3 (13.9–18.8)	96.8	Ref	Ref	<0.01
Nonretrospective^b^	10	5207	652	14.1 (11.5–16.9)	84.9	−0.028 (−.105 to .049)	.474	…
Sample size^c^								
<500	49	14 197	2360	15.7 (13.6–18.0)	92.6	Ref	Ref	<0.01
≥500	20	18 853	3435	16.6 (12.4–21.3)	98.5	0.012 (−.046 to .071)	.678	…
Year								
2015 or after	28	10 883	1889	16.1 (12.6–19.8)	96.2	Ref	Ref	<0.01
Before 2015	41	22 167	3906	15.9 (13.3–18.7)	96.7	−0.002 (−.057 to .053)	.938	…
Total	69	33 050	5795	16.0 (13.9–18.2)	96.5	…		…

Abbreviation: CI, confidence interval.

^a^Other refers to multiple surgeries and a specific type of surgery, other than orthopedic or abdominal surgeries; these were reported in a small number of studies.

^b^Study type comprises prospective or surveillance.

^c^The number of all *Pseudomonas aeruginosa* isolates.

### Proportions of Antimicrobial Resistance

**Figure 5.**
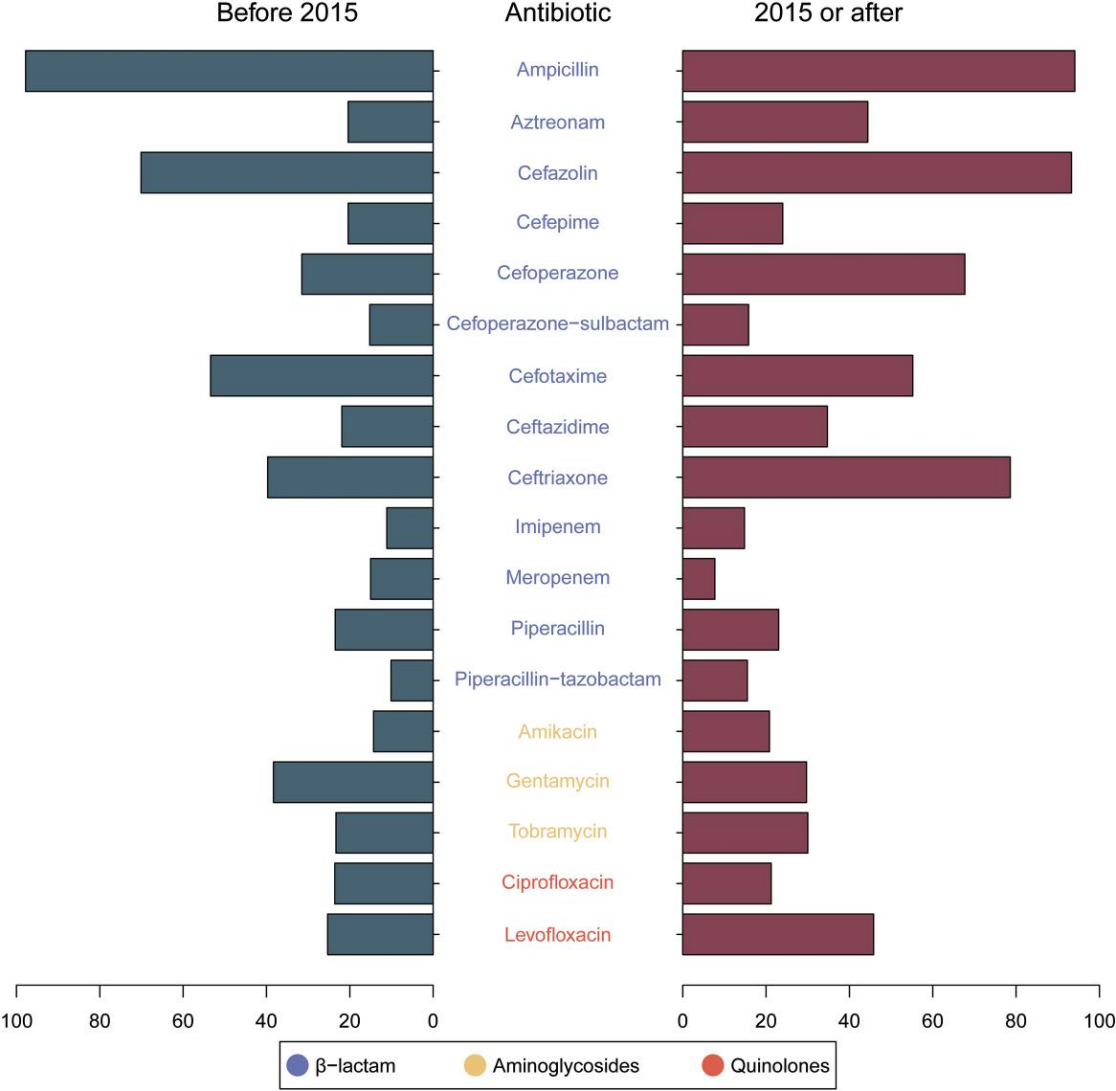
Antibiotic resistance rates for *Pseudomonas aeruginosa* for different subgroups of years.

We further analyzed the resistance of *P aeruginosa* to different antibiotics, and 54 of the 72 papers reported *P aeruginosa* resistance to 69 antibiotics. Among them, 18 antibiotics were reported in 10 or more studies (Table 3, Figure 5). We performed a meta-analysis of these antibiotics and compared the antimicrobial resistance rates for *P aeruginosa* before and after 2015. The results indicated that compared with that reported in the studies conducted before 2015 (31.5% [95% CI, 20.2%–43.8%]), the rate of antimicrobial resistance of *P aeruginosa* to cefoperazone (67.7% [95% CI, 54.6%–79.6%]) was significantly higher in the studies conducted after 2015 (*R*^2^ = 42.75). Similarly, the rate of resistance to ceftriaxone was significantly higher after 2015 (*R*^2^ = 42.27)—that is, 39.7% (95% CI, 27.6%–52.5%) before 2015 and 78.6% (95% CI, 61.5%–91.9%) in 2015 and after. After 2015, there was also a notable increase in the resistance rate to levofloxacin (*R*^2^ = 12.49) and aztreonam (*R*^2^ = 10.88). In addition, the resistance of *P aeruginosa* to cefazolin and ceftazidime showed increasing trends, but there was no significant difference between the subgroups. Notably, the rate of resistance of *P aeruginosa* to ampicillin and cefazolin exceeded 90.0%. The resistance rates of *P aeruginosa* in different regions and types of hospitals are detailed in Supplementary Tables 3 and 4.

**Table 3.** Results of the Combined Proportions of Drug-Resistant *Pseudomonas aeruginosa* at Different Times

Drug	Year	Proportions of Resistant *Pseudomonas aeruginosa*					Univariate Meta-regression		
		Studies	*Pseudomonas aeruginosa*	Resistant *Pseudomonas aeruginosa*	Estimate, %	*I* ^2^	Coefficient (95% CI)	*P* Value	*R* ^2^
Ampicillin	2015 or after	13	889	836	94.1 (88.0–98.3)	88.3	Ref	Ref	<0.01
	Before 2015	9	592	567	97.8 (92.4–100.0)	87.8	0.087 (−.097 to .271)	.353	…
Aztreonam	2015 or after	15	1029	489	44.4 (18.5–72.0)	98.7	Ref	Ref	10.88
	Before 2015	13	1727	315	20.4 (12.9–29.0)	92.7	−0.257 (−.500 to −.013)	.039	…
Cefazolin	2015 or after	9	725	652	93.3 (85.3–98.6)	91.0	Ref	Ref	10.66
	Before 2015	8	518	349	70.1 (35.6–95.4)	98.4	−0.306 (−.661 to .048)	.090	…
Cefepime	2015 or after	14	997	295	24.0 (9.1–43.0)	97.4	Ref	Ref	<0.01
	Before 2015	21	2519	501	20.4 (15.2–26.1)	89.7	−0.040 (−.205 to .124)	.631	…
Cefoperazone	2015 or after	4	299	184	67.7 (54.6–79.6)	76.1	Ref	Ref	42.75
	Before 2015	8	625	188	31.5 (20.2–43.8)	87.5	−0.370 (−.629 to −.110)	.005	…
Cefoperazone-sulbactam	2015 or after	6	524	88	15.8 (5.8–29.3)	92.3	Ref	Ref	<0.01
	Before 2015	13	1930	206	15.2 (9.6–21.8)	91.3	−0.007 (−.166 to .152)	.933	…
Cefotaxime	2015 or after	6	365	157	55.2 (37.4–72.4)	89.3	Ref	Ref	<0.01
	Before 2015	10	766	352	53.4 (35.2–71.1)	95.8	−0.025 (−.302 to .252)	.860	…
Ceftazidime	2015 or after	17	1171	444	34.7 (19.0–52.3)	97.2	Ref	Ref	4.91
	Before 2015	26	3201	727	21.9 (16.5–27.9)	92.4	−0.137 (−.283 to .010)	.067	…
Ceftriaxone	2015 or after	12	711	530	78.6 (61.5–91.9)	95.6	Ref	Ref	42.27
	Before 2015	8	764	309	39.7 (27.6–52.5)	91.2	−0.398 (−.608 to −.189)	.000	…
Imipenem	2015 or after	22	1481	369	14.8 (5.2–27.6)	97.2	Ref	Ref	<0.01
	Before 2015	28	3129	434	11.1 (6.7–16.4)	93.6	−0.055 (−.183 to .074)	.405	…
Meropenem	2015 or after	9	588	56	7.7 (1.9–16.3)	89.9	Ref	Ref	6.33
	Before 2015	17	1558	290	15.0 (9.7–21.1)	88.9	0.112 (−.033 to .256)	.130	…
Piperacillin	2015 or after	11	673	161	23.0 (8.0–42.4)	96.4	Ref	Ref	<0.01
	Before 2015	17	2289	595	23.5 (18.7–28.6)	82.9	−0.004 (−.176 to .169)	.968	…
Piperacillin-tazobactam	2015 or after	7	429	78	15.5 (6.4–27.4)	88.4	Ref	Ref	0.19
	Before 2015	15	1821	188	10.1 (5.8–15.4)	88.2	−0.079 (−.224 to .065)	.281	…
Amikacin	2015 or after	19	1363	385	20.8 (6.7–39.8)	98.2	Ref	Ref	<0.01
	Before 2015	22	2527	386	14.3 (9.6–19.7)	90.9	−0.081 (−.276 to .113)	.412	…
Gentamycin	2015 or after	17	1093	353	29.7 (13.4–49.0)	97.6	Ref	Ref	<0.01
	Before 2015	22	2892	1020	38.3 (30.0–47.1)	95.0	0.090 (−.106 to .286)	.368	…
Tobramycin	2015 or after	12	934	342	30.0 (7.8–58.6)	98.7	Ref	Ref	<0.01
	Before 2015	7	797	200	23.3 (14.6–33.3)	88.8	−0.075 (−.402 to .251)	.651	…
Ciprofloxacin	2015 or after	17	1079	198	21.2 (13.8–29.7)	89.6	Ref	Ref	<0.01
	Before 2015	24	3050	762	23.6 (16.5–31.5)	95.3	0.027 (−.105 to .159)	.691	…
Levofloxacin	2015 or after	15	1124	545	45.8 (25.8–66.4)	97.9	Ref	Ref	12.49
	Before 2015	21	2713	612	25.3 (18.8–32.4)	92.9	−0.210 (−.382 to −.038)	.017	…

Abbreviation: CI, confidence interval.

## DISCUSSION

In this study, we conducted a meta-analysis of the epidemiological characteristics and antimicrobial resistance of *P aeruginosa* in SSIs in China. Compared with the data reported by the National Nosocomial Infection Surveillance System [17], the overall proportion of *P aeruginosa* among SSIs in this study was higher. In addition, our results showed that the rate of resistance of *P aeruginosa* to a variety of antibiotics (cefoperazone, ceftriaxone, levofloxacin, and aztreonam) was significantly increased.

The total proportion of *P aeruginosa* among SSIs (16.0%) was lower than the findings reported in Malaysia (26.25%) but significantly higher than the data reported by the National Nosocomial Infection Surveillance System (8.9%) [17, 100]. We analyzed the reasons for the discrepancy between our results and the data reported by the National Nosocomial Infection Surveillance System. We believe that this discrepancy may be caused by the following reasons. First, the data reported by the National Nosocomial Infection Surveillance System include all nosocomial infections. Among them, the number of bacteria isolated from SSIs was 1638, far less than the 33 050 strains isolated from SSIs in our study. In addition, the 69 studies included in our study involved >69 hospitals distributed in 24 different provinces, including tertiary hospitals and nontertiary hospitals. Second, the studies included in this study were all conducted after 2010, and some of the studies (28) were even completed after 2015. However, the data reported by the National Nosocomial Infection Surveillance System were collected in 2014. Over time, the proportions of *P aeruginosa* among SSI samples may fluctuate, leading to a difference between the results of this study and the data collected by surveillance systems.

In addition, the results of our study provide more detailed information on SSIs caused by *P aeruginosa*. Groupings were made by type of surgery, hospital level, and different regions and provinces, and then, the proportions of *P aeruginosa* among patients with SSIs in the different subgroups were analyzed. Subgroup analysis showed that the proportions of *P aeruginosa* varied between different types of surgery, with higher rates in orthopedic surgery (18.3%) and abdominal surgery (17.3%) and lower rates in other types of surgery (11.3%). The results indicated that compared with other types of surgery, patients undergoing orthopedic and abdominal surgery were more likely to have SSIs caused by *P aeruginosa*. This finding is consistent with the results of previous studies, suggesting that *P aeruginosa* is one of the main pathogenic causes of SSIs after orthopedic surgery and abdominal surgery and that *P aeruginosa* should be suspected if a SSI occurs after orthopedic surgery and abdominal surgery [101–103]. The proportions of *P aeruginosa* in SSI samples in different regions were different. Among them, the proportion of *P aeruginosa* in SSI samples was highest for the central region (18.6%); importantly, most of the studies from the central region were orthopedic surgery–related or abdominal surgery–related studies.

*Pseudomonas aeruginosa* is resistant to a large number of antimicrobial drugs. It is particularly difficult to treat infections caused by this microorganism, and a delay in treatment can lead to increased mortality. Empirical treatment is dependent on microbiological test results. However, the high resistance of pathogenic bacteria to antibiotics may increase the possibility of inappropriate empirical treatment, resulting in poor clinical treatment outcomes and increasing the financial burden on patients. Therefore, in addition to taking appropriate antimicrobial treatment measures in a timely manner and following the guidelines for the use of antibiotics, it is also necessary to design an empirical treatment plan based on the results of antimicrobial susceptibility tests [104, 105]. In addition to intrinsic antimicrobial resistance, *P aeruginosa* can also acquire antimicrobial resistance through other known antimicrobial resistance mechanisms and then develop into MDR bacteria or pandrug-resistant bacteria, leading to life-threatening serious infections [106–108]. In this study, variations exist in the antimicrobial resistance rates of *P aeruginosa* across different regions. In particular, the central region exhibits generally higher resistance rates compared to the other 2 regions, notably for antimicrobial agents such as amikacin, ciprofloxacin, and levofloxacin. These findings are consistent with the higher detection proportion of *P aeruginosa* in the central region. The rate of antimicrobial resistance of *P aeruginosa* to the carbapenem antibiotic meropenem (12.2%) is comparable to the rate of antimicrobial resistance reported by the Chinese Antimicrobial Resistance Surveillance System in 2020 (14.4%), but the overall trend of resistance is decreasing. The antimicrobial resistance rate before 2015 was 15.0%, and the antimicrobial resistance rate after 2015 was 7.7%. In this study, the resistance of *P aeruginosa* to meropenem was comparable to that reported in Uganda (14.0%) but significantly lower than that reported in southwestern Iran (49.5%) and Taiwan (73.2%), a finding that may be related to whether the application of antibiotics was reasonable [109–111].

*Pseudomonas aeruginosa* produces β-lactamase, which is the main cause of its resistance to carbapenem antibiotics. Such strains of *P aeruginosa* are not only resistant to carbapenem antibiotics but may also develop significant resistance to other β-lactam antibiotics [112, 113]. In this study, the rates of resistance of *P aeruginosa* to cefoperazone (31.5% before 2015 and 67.7% after 2015), ceftriaxone (39.7% before 2015 and 78.6% after 2015), and aztreonam (20.4% before 2015 and 40.4% after 2015) significantly increased after 2015. The possible reason is that a large amount of these 3 antibiotics were used in clinical treatments, resulting in an increase in the resistance of *P aeruginosa* to these 3 antibiotics. According to data from the National Nosocomial Infection Surveillance System, the average rate of use of antimicrobial drugs in hospitalized patients in China was 50.5%, which is comparable to that of India (57%) [114]. However, this rate is considerably higher than that reported in the Netherlands (30.9%), the United Kingdom (34.7%), and Canada (36.3%) [18]. Clinical practitioners in China demonstrate a proclivity toward the use of broad-spectrum antimicrobial agents or combination therapy when treating infections [115]. The above results suggest that clinicians need to avoid the abovementioned antibiotics, which produce high antimicrobial resistance in clinical practice. In the follow-up prevention and treatment of infections, the use of antimicrobial drugs should be more strictly monitored, and antimicrobial drug sensitivity tests should be used to guide treatment [18]. Notably, when analyzing the antimicrobial resistance of *P aeruginosa* in this study, it was found to be resistant to cefazolin (70.1% before 2015 and 93.3% after 2015) and ampicillin (97.8% before 2015 and 94.1% after 2015), a finding that is consistent with the results of a study in Ethiopia [116]. Despite not being recommended by the Clinical and Laboratory Standards Institute guidelines, research has continued to utilize them for susceptibility testing over the past decade. Our study confirms their ineffectiveness for susceptibility testing of *P aeruginosa*, offering valuable guidance for future clinical microbiological testing.

Aminoglycoside antibiotics are also commonly used antimicrobial drugs for the treatment of *P aeruginosa* infection. In this study, the resistance of *P aeruginosa* to aminoglycoside antibiotics, represented by amikacin, tobramycin, and gentamycin, was low. There was also a decreasing trend in the resistance to gentamycin (38.3% before 2015 and 29.7% after 2015). Quinolone antibiotics are the most recently discovered class of drugs and are active against gram-negative bacteria [117]. In this study, the antimicrobial resistance of *P aeruginosa* to quinolone antibiotics, represented by levofloxacin and ciprofloxacin, may be different because of nonrational use and because resistance to levofloxacin (25.3% before 2015 and 45.8% after 2015) significantly increased. However, the resistance to ciprofloxacin (23.6% before 2015 and 21.2% after 2015) showed a slight downward trend.

This study has the following limitations. First, the heterogeneity among the included studies was considerable, and the subgroup analysis and meta-regression analysis could not fully explain the source of the heterogeneity. Second, because small-sample studies are prone to producing accidental results, the analysis excluded studies with a small number of bacterial strains isolated from SSIs, resulting in a lack of data on the proportions of *P aeruginosa* in some provinces. In addition, the exclusion of this type of study meant that most of the surgical procedures included in the study were orthopedic and abdominal surgeries. The proportions of *P aeruginosa* infection were high in these 2 types of surgeries, potentially leading to a high estimate. Third, the majority of the regions included in the study were the central and eastern coastal areas, and the monitoring of surgical wound infections was more common in these areas. Therefore, the estimated proportions of *P aeruginosa* cannot represent the overall situation of SSIs in China.

## CONCLUSIONS

In summary, the proportions of *P aeruginosa* and the susceptibility to antimicrobial drugs vary with region and time and need to be monitored at all times. Compared with the data reported by the Chinese Antimicrobial Resistance Surveillance System, the proportion of *P aeruginosa* among SSIs obtained in this study was higher. Therefore, it is necessary to carry out long-term monitoring to understand the actual proportion and antimicrobial resistance of *P aeruginosa* among SSIs and to develop appropriate healthcare mechanisms. *Pseudomonas aeruginosa* isolated from surgical sites has high antimicrobial resistance to ampicillin and cefazolin, and antimicrobial resistance to cefoperazone, ceftriaxone, levofloxacin, and aztreonam has significantly increased. We therefore recommend initiating appropriate infection prevention measures, strengthening existing antimicrobial stewardship programs, and conducting regular antimicrobial surveillance to prevent antimicrobial-resistant *P aeruginosa* in hospitals.

## Supplementary Material

ofad647_Supplementary_Data

## Supplementary Data

Supplementary materials are available at *Open Forum Infectious Diseases* online. Consisting of data provided by the authors to benefit the reader, the posted materials are not copyedited and are the sole responsibility of the authors, so questions or comments should be addressed to the corresponding author.

## Notes

***Patient consent.*** This study does not include factors necessitating patient consent.

***Financial support*.** This project was supported by the National Science Foundation of China (number 32100116) and the Center for Disease Control and Prevention of the Central Theater Command (number jkzx2022-02).
