# Supplementary material for: Proportions of Pseudomonas aeruginosa and Antimicrobial-Resistant P aeruginosa Among Patients With Surgical Site Infections in China: A Systematic Review and Meta-analysis
Source: Open Forum Infect Dis. 2023 Dec 18;11(2):ofad647. doi: 10.1093/ofid/ofad647 (PMC10847825; doi:10.1093/ofid/ofad647)
Supplement: ofad647_Supplementary_Data [file ofad647_supplementary_data.docx]

**SUPPLEMENTARY MATERIALS**

**Table 1. Search strategy used in each database**

PubMed

| Step | Query | Results |
| --- | --- | --- |
| 1 | ((Surgery) AND ((((("Cross Infection"[Mesh]) OR (cross infection)) OR (Nosocomial Infection)) OR (hospital infection)) OR (infection*))) AND (((((("Wound Infection"[Mesh]) OR "Surgical Wound"[Mesh]) OR (Wound Infection)) OR (Surgical Wound)) OR (postoperative wound infection)) OR (surgical site infection)) | 77129 |
| 2 | ("Surgical Wound Infection"[Mesh]) OR (Surgical Wound Infection) | 59207 |
| 3 | Step 1 OR Step 2 | 84714 |
| 4 | ("Pseudomonas aeruginosa"[Mesh]) OR (Pseudomonas aeruginosa) | 80910 |
| 5 | ((("China"[Mesh] OR "Taiwan"[Mesh]) OR (China)) OR (Chinese)) OR (Taiwan) | 3207477 |
| 6 | Step 3 AND Step 4 AND Step 5 | 106 |

Embase

| Step | Query | Results |
| --- | --- | --- |
| 1 | ('surgery'/exp OR surgery:ti,ab,kw) AND ('cross infection'/exp OR 'surgical infection'/exp OR 'hospital infection'/exp OR 'infection'/exp OR 'surgical infection':ti,ab,kw OR 'cross infection':ti,ab,kw OR 'hospital infection':ti,ab,kw OR infection*:ti,ab,kw OR 'nosocomial infection':ti,ab,kw) AND ('wound infection'/exp OR 'surgical wound'/exp OR 'wound infection':ti,ab,kw OR 'surgical wound':ti,ab,kw OR 'postoperative wound infection':ti,ab,kw OR 'surgical site infection':ti,ab,kw) | 74022 |
| 2 | 'pseudomonas aeruginosa'/exp OR 'pseudomonas aeruginosa':ti,ab,kw | 140774 |
| 3 | 'china'/exp OR 'chinese'/exp OR 'taiwan'/exp OR china:ti,ab,kw OR chinese:ti,ab,kw OR taiwan:ti,ab,kw | 771045 |
| 4 | Step 1 AND Step 2 AND Step 3 | 29 |

Web of Science

| Step | Query | Results |
| --- | --- | --- |
| 1 | ALL=(Surgery)AND (((((ALL=(infection*)) OR ALL=(surgical infection)) OR ALL=(cross infection)) OR ALL=(Nosocomial Infection)) OR ALL=(hospital infection)) AND (((((ALL=(Wound Infection)) OR ALL=(postoperative wound infection)) OR ALL=(Surgical Wound)) OR ALL=(surgical site infection)) OR ALL=(Surgical Wound Infection)) | 43037 |
| 2 | ALL=(Pseudomonas aeruginosa) | 85874 |
| 3 | ((ALL=(China)) OR ALL=(Chinese)) OR ALL=(Taiwan) | 8394098 |
| 4 | Step 1 AND Step 2 AND Step 3 | 94 |

Chinese Biomedical Literature Database (CBM)

| Step | Query | Results |
| --- | --- | --- |
| 1 | "手术"[常用字段:智能] OR "术后"[常用字段:智能] OR "外科"[常用字段:智能] | 5052022 |
| 2 | "伤口感染"[不加权:扩展] OR "伤口感染"[常用字段:智能] OR "切口感染"[常用字段:智能] OR "创面感染"[常用字段:智能] OR "部位感染"[常用字段:智能] OR "腔隙感染"[常用字段:智能] OR "器官感染"[常用字段:智能] | 124648 |
| 3 | Step 1 AND Step 2 | 102299 |
| 4 | "外科伤口感染"[不加权:扩展] | [56270](javascript:historyLink(') |
| 5 | Step 3 OR Step 4 | [102785](javascript:historyLink('() |
| 6 | "铜绿假单胞菌"[不加权:扩展] OR "铜绿假单胞菌"[常用字段:智能] OR "绿脓假单胞菌"[常用字段:智能] | [97348](javascript:historyLink(') |
| 7 | Step 5 AND Step 6 | 726 |

China National Knowledge Infrastructure (CNKI)

| Step | Query | Results |
| --- | --- | --- |
| 1 | ( SU % "手术"+"术后"+"外科") AND (SU % "伤口感染"+"切口感染"+"创面感染"+"部位感染"+"腔隙感染"+"器官感染") AND (SU % "铜绿假单胞菌"+"绿脓假单胞菌") | 116 |

Wanfang Database

| Step | Query | Results |
| --- | --- | --- |
| 1 | 主题:(手术) OR 主题:(术后) OR 主题:(外科) | 7435637 |
| 2 | 主题:(伤口感染) OR 主题:(切口感染) OR 主题:(创面感染) OR 全部:(部位感染) OR 全部:(腔隙感染) OR 全部:(器官感染) | 214371 |
| 3 | 主题:(铜绿假单胞菌) OR 主题:(绿脓假单胞菌) | 34764 |
| 4 | Step 1 AND Step 2 AND Step 3 | 1453 |

Weipu Database

| Step | Query | Results |
| --- | --- | --- |
| 1 | U=(手术 OR 术后 OR 外科) | [3402623](https://gffyb419fc6405fe94a31s999cwvp09xxc60f6fffb.res.gxlib.org.cn/Qikan/search/index?LngMySearHistoryIdGuid=800ed8b2-a56d-4c4f-a02f-6964d58e3680&from=Qikan_Article_History) |
| 2 | U=(伤口感染 OR 切口感染 OR 创面感染 OR 部位感染 OR 腔隙感染 OR 器官感染) | [115088](https://gffyb419fc6405fe94a31s999cwvp09xxc60f6fffb.res.gxlib.org.cn/Qikan/search/index?LngMySearHistoryIdGuid=cd1f066e-812d-4e73-af72-3c3d3d5ccc70&from=Qikan_Article_History) |
| 3 | U=(铜绿假单胞菌 OR 绿脓假单胞菌) | [28622](https://gffyb419fc6405fe94a31s999cwvp09xxc60f6fffb.res.gxlib.org.cn/Qikan/search/index?LngMySearHistoryIdGuid=d8e40392-3c77-4a13-b6ab-9badfa8513ea&from=Qikan_Article_History) |
| 4 | Step 1 AND Step 2 AND Step 3 | 1181 |

**Table 2. Quality evaluation results for the included studies**

| Study ID | Specified diagnostic criteria of original diseases | Specified diagnostic criteria of SSIs | Specified criteria of inclusion | Case source | Case selection method | Specified test for pathogenic bacteria | Pathogenic test for all included cases | Study type | Total scores |
| --- | --- | --- | --- | --- | --- | --- | --- | --- | --- |
| Chen 2012 | Yes | Unclear | Yes | Yes | Yes | NO | Yes | Retrospective | 5 |
| Gao 2013 | Unclear | Unclear | Unclear | Yes | Yes | Yes | Yes | Retrospective | 4 |
| Han 2013 | Unclear | Unclear | Unclear | Yes | Unclear | Unclear | Unclear | Retrospective | 1 |
| You 2013 | Unclear | Unclear | Unclear | Yes | Unclear | Yes | Unclear | Retrospective | 2 |
| Zhao 2013 | Unclear | NO | Unclear | Yes | Yes | Unclear | Yes | Retrospective | 3 |
| Feng 2014 | Unclear | NO | Unclear | Yes | Unclear | Yes | Yes | Retrospective | 3 |
| Gao 2014 | Unclear | Yes | Unclear | Yes | Yes | NO | Yes | Monitoring | 5 |
| Guo 2014 | Unclear | Unclear | Unclear | Yes | Yes | Yes | Unclear | Retrospective | 3 |
| Huang 2014 | Unclear | Unclear | Unclear | Yes | Unclear | Yes | Yes | Retrospective | 3 |
| Liu 2014 | Unclear | Yes | Unclear | Unclear | Unclear | Yes | Yes | Retrospective | 3 |
| Mo 2014 | Unclear | Unclear | Unclear | Yes | Yes | Yes | Yes | Prospective | 5 |
| Zheng 2014 | Unclear | Unclear | Unclear | Yes | Unclear | Unclear | Yes | Retrospective | 2 |
| Chen 2015 | Unclear | Unclear | Unclear | Yes | Unclear | Yes | Yes | Retrospective | 3 |
| Lou 2015 | Unclear | Unclear | Unclear | Yes | Yes | Yes | Yes | Retrospective | 4 |
| Ran 2015 | Unclear | Yes | Unclear | Yes | Yes | NO | Yes | Prospective | 5 |
| Su 2015 | Unclear | Unclear | Unclear | Yes | Yes | Yes | Yes | Monitoring | 5 |
| Wang 2015 | Unclear | Unclear | Unclear | Yes | Unclear | Yes | Yes | Retrospective | 3 |
| Xie 2015 | Unclear | Unclear | Unclear | Yes | Yes | Unclear | Yes | Monitoring | 4 |
| Yang 2015 | Unclear | Yes | Unclear | Yes | Yes | Yes | Yes | Retrospective | 5 |
| Yao 2015 | Unclear | Yes | Unclear | Yes | Unclear | Yes | Yes | Monitoring | 5 |
| Zhong 2015 | Unclear | Yes | Unclear | Yes | Yes | NO | Yes | Retrospective | 4 |
| Dong 2016 | Unclear | Unclear | Unclear | Yes | Yes | Yes | Yes | Retrospective | 4 |
| Hu 2016 | Unclear | Yes | Unclear | Yes | Yes | Yes | Yes | Retrospective | 5 |
| Peng 2016 | Unclear | Yes | Unclear | Yes | Yes | Yes | Yes | Retrospective | 5 |
| Wang 2016 | Unclear | Unclear | Unclear | Yes | Unclear | Yes | Yes | Retrospective | 3 |
| Wang 2016 | Unclear | Unclear | Unclear | Yes | Unclear | NO | Unclear | Retrospective | 1 |
| Yang 2016 | Unclear | Yes | Unclear | Yes | Yes | Yes | Yes | Retrospective | 5 |
| Yu 2016 | Unclear | Unclear | Unclear | Yes | Unclear | Yes | Yes | Retrospective | 3 |
| Chen 2017 | Unclear | Yes | Unclear | Yes | Yes | Yes | Yes | Retrospective | 5 |
| Jin 2017 | Unclear | Yes | Unclear | Yes | Unclear | Yes | Yes | Retrospective | 4 |
| Li 2017 | Yes | Unclear | Yes | Yes | Unclear | Yes | Yes | Retrospective | 5 |
| Liu 2017 | Unclear | Yes | Unclear | Yes | Yes | NO | Yes | Retrospective | 4 |
| Liu 2017 | Unclear | Yes | Yes | Yes | Unclear | Unclear | Yes | Retrospective | 4 |
| Luo 2017 | Unclear | Unclear | Unclear | Yes | Unclear | Unclear | Yes | Retrospective | 2 |
| Wan 2017 | Unclear | Yes | Unclear | Yes | Unclear | Yes | Yes | Retrospective | 4 |
| Wu 2017 | Unclear | Yes | Unclear | Yes | Yes | Yes | Yes | Retrospective | 5 |
| Wu 2017 | Unclear | Yes | Unclear | Yes | Yes | Yes | Yes | Retrospective | 5 |
| Ren 2018 | Unclear | Unclear | Unclear | Yes | Unclear | Yes | Yes | Retrospective | 3 |
| Sun 2018 | Unclear | Unclear | Unclear | Yes | Unclear | Yes | Yes | Retrospective | 3 |
| Xie 2018 | Unclear | Unclear | Unclear | Yes | Unclear | Yes | Yes | Retrospective | 3 |
| Yang 2018 | Unclear | Yes | Unclear | Yes | Unclear | Yes | Yes | Retrospective | 4 |
| Zhou 2018 | Unclear | Yes | Unclear | Yes | Unclear | Unclear | Yes | Retrospective | 3 |
| Zhou 2018 | Unclear | NO | Unclear | Yes | Yes | Yes | Yes | Retrospective | 4 |
| Gao 2019 | Unclear | Yes | Yes | Yes | Yes | Yes | Yes | Monitoring | 7 |
| Hu 2019 | Unclear | Unclear | Yes | Yes | Unclear | Yes | Yes | Retrospective | 4 |
| Liang 2019 | Unclear | Unclear | Yes | Yes | Unclear | Unclear | Yes | Retrospective | 3 |
| Wang 2019 | Unclear | Yes | Yes | Yes | Unclear | Yes | Yes | Retrospective | 5 |
| Yu 2019 | Unclear | Yes | Yes | Yes | Unclear | Yes | Yes | Retrospective | 5 |
| Chai 2020 | Unclear | Unclear | Unclear | Yes | Unclear | Yes | Yes | Retrospective | 3 |
| Gong 2020 | Unclear | Unclear | Unclear | Yes | Yes | Yes | Yes | Retrospective | 4 |
| Huang 2020 | Unclear | Unclear | Unclear | Yes | Yes | Yes | Yes | Retrospective | 4 |
| Lin 2020 | Unclear | Unclear | Unclear | Yes | Yes | Yes | Yes | Monitoring | 5 |
| Pan 2020 | Unclear | Unclear | Unclear | Yes | Yes | Yes | Unclear | Retrospective | 3 |
| Wang 2020 | Yes | Unclear | Yes | Yes | Unclear | Unclear | Yes | Retrospective | 4 |
| Zhao 2020 | Unclear | Yes | Unclear | Yes | Unclear | Yes | Yes | Retrospective | 4 |
| Dong 2021 | Unclear | Unclear | Unclear | Yes | Unclear | Unclear | Unclear | Retrospective | 1 |
| Wang 2021 | Unclear | Unclear | Unclear | Yes | Yes | Yes | Yes | Retrospective | 4 |
| Wang 2021 | Unclear | Unclear | Unclear | Yes | Yes | Yes | Yes | Monitoring | 5 |
| Xu 2021 | Unclear | Unclear | Unclear | Yes | Unclear | Unclear | Yes | Retrospective | 2 |
| Yang 2021 | Unclear | Unclear | Yes | Yes | Unclear | Yes | Yes | Retrospective | 4 |
| Ye 2021 | Unclear | Unclear | Unclear | Yes | Unclear | Yes | Yes | Retrospective | 3 |
| Yu 2021 | Unclear | Unclear | Unclear | Yes | Yes | Yes | Yes | Retrospective | 4 |
| Zheng 2021 | Unclear | Unclear | Yes | Yes | Yes | Unclear | Yes | Retrospective | 4 |
| Chen 2022 | Unclear | Unclear | Yes | Yes | Yes | Yes | Yes | Retrospective | 5 |
| Li 2022 | Unclear | Yes | Yes | Yes | Unclear | Yes | Yes | Retrospective | 5 |
| Ma 2022 | Unclear | Unclear | Unclear | Yes | Yes | Yes | Yes | Retrospective | 4 |
| Sun 2022 | Unclear | Yes | Unclear | Unclear | Yes | Yes | Yes | Monitoring | 5 |
| Wang 2022 | Yes | Unclear | Yes | Yes | Yes | Yes | Yes | Retrospective | 6 |
| Xie 2022 | Unclear | Yes | Yes | Yes | Unclear | Yes | Yes | Retrospective | 5 |
| Cao 2023 | Yes | Unclear | Yes | Yes | Yes | Yes | Yes | Retrospective | 6 |
| Han 2023 | Unclear | Unclear | Yes | Yes | Yes | Yes | Yes | Retrospective | 5 |
| Zhang 2023 | Unclear | Unclear | Yes | Yes | Yes | Unclear | Yes | Retrospective | 4 |

**Table 3. Results of the combined proportions of drug-resistant *Pseudomonas aeruginosa* at different hospital level**

|  | Hospital level | Proportions of resistant *Pseudomonas aeruginosa* | | | | |  | Univariate meta-regression | | |
| --- | --- | --- | --- | --- | --- | --- | --- | --- | --- | --- |
|  |  | Studies | *Pseudomonas aeruginosa* | Resistant *Pseudomonas aeruginosa* | Estimate | *I*^2^ |  | Coefficient (95% CI) | p value | *R*^2^ |
| Ampicillin | Tertiary | 15 | 1048 | 993 | 95.0% (89.3-98.8) | 90.3 |  | Ref | Ref | <0.01 |
|  | Non-tertiary | 7 | 433 | 410 | 97.3% (92.1-100.0) | 77.8 |  | 0.056 (-0.143 to 0.255) | 0.582 |  |
| Aztreonam | Tertiary | 21 | 2416 | 595 | 28.1% (15.7-42.5) | 98.0 |  | Ref | Ref | 2.65 |
|  | Non-tertiary | 7 | 340 | 209 | 46.1% (10.3-84.4) | 98.1 |  | 0.188 (-0.106 to 0.482) | 0.211 |  |
| Cefazolin | Tertiary | 13 | 856 | 700 | 86.2% (68.2-97.7) | 97.5 |  | Ref | Ref | <0.01 |
|  | Non-tertiary | 4 | 387 | 301 | 76.1% (44.5-97.1) | 97.3 |  | -0.127 (-0.577 to 0.324) | 0.582 |  |
| Cefepime | Tertiary | 25 | 3035 | 683 | 23.0% (15.4-31.7) | 96.2 |  | Ref | Ref | <0.01 |
|  | Non-tertiary | 10 | 481 | 113 | 18.0% (7.5-31.5) | 90.8 |  | -0.060 (-0.240 to 0.120) | 0.512 |  |
| Cefoperazone | Tertiary | 9 | 699 | 246 | 39.0% (25.0-54.0) | 92.5 |  | Ref | Ref | 1.71 |
|  | Non-tertiary | 3 | 225 | 126 | 59.5% (35.4-81.5) | 86.1 |  | 0.201 (-0.163 to 0.565) | 0.279 |  |
| Cefoperazone-sulbactam | Tertiary | 13 | 2030 | 228 | 15.2% (9.0-22.5) | 93.6 |  | Ref | Ref | <0.01 |
|  | Non-tertiary | 6 | 424 | 66 | 15.9% (9.3-23.9) | 71.8 |  | 0.014 (-0.147 to 0.175) | 0.866 |  |
| Cefotaxime | Tertiary | 9 | 665 | 263 | 44.4% (33.8-55.2) | 84.8 |  | Ref | Ref | 8.96 |
|  | Non-tertiary | 7 | 466 | 246 | 64.9% (38.5-87.4) | 96.7 |  | 0.189 (-0.060 to 0.438) | 0.138 |  |
| Ceftazidime | Tertiary | 31 | 3727 | 948 | 26.7% (19.3-34.8) | 96.2 |  | Ref | Ref | <0.01 |
|  | Non-tertiary | 12 | 645 | 223 | 25.8% (12.8-41.3) | 93.9 |  | -0.010 (-0.177 to 0.157) | 0.908 |  |
| Ceftriaxone | Tertiary | 15 | 1131 | 688 | 68.3% (50.6-83.8) | 97.1 |  | Ref | Ref | 3.03 |
|  | Non-tertiary | 5 | 344 | 151 | 48.9% (28.4-69.7) | 92.2 |  | -0.190 (-0.493 to 0.113) | 0.218 |  |
| Imipenem | Tertiary | 37 | 3838 | 594 | 12.8% (8.4-17.9) | 94.1 |  | Ref | Ref | <0.01 |
|  | Non-tertiary | 13 | 772 | 209 | 12.0% (0.5-33.3) | 97.9 |  | -0.007 (-0.154 to 0.141) | 0.931 |  |
| Meropenem | Tertiary | 20 | 1723 | 296 | 12.7% (7.9-18.4) | 89.7 |  | Ref | Ref | <0.01 |
|  | Non-tertiary | 6 | 423 | 50 | 10.7% (1.9-24.4) | 92.4 |  | -0.031 (-0.203 to 0.140) | 0.719 |  |
| Piperacillin | Tertiary | 22 | 2683 | 675 | 22.1% (15.5-29.4) | 93.7 |  | Ref | Ref | <0.01 |
|  | Non-tertiary | 6 | 279 | 81 | 26.4% (12.7-42.7) | 87.1 |  | 0.050 (-0.157 to 0.256) | 0.637 |  |
| Piperacillin-tazobactam | Tertiary | 16 | 1995 | 235 | 13.0% (7.8-19.1) | 91.2 |  | Ref | Ref | <0.01 |
|  | Non-tertiary | 6 | 255 | 31 | 8.3% (2.6-16.3) | 69.6 |  | -0.077 (-0.235 to 0.080) | 0.336 |  |
| Amikacin | Tertiary | 30 | 3181 | 616 | 16.9% (9.0-26.6) | 97.5 |  | Ref | Ref | <0.01 |
|  | Non-tertiary | 11 | 709 | 155 | 17.9% (8.2-30.0) | 92.4 |  | 0.012 (-0.209 to 0.233) | 0.916 |  |
| Gentamycin | Tertiary | 28 | 3437 | 1067 | 30.6% (21.9-39.9) | 96.7 |  | Ref | Ref | 2.44 |
|  | Non-tertiary | 11 | 548 | 306 | 45.1% (29.0-61.8) | 92.8 |  | 0.146 (-0.069 to 0.361) | 0.184 |  |
| Tobramycin | Tertiary | 16 | 1353 | 445 | 29.0% (12.2-49.3) | 98.2 |  | Ref | Ref | <0.01 |
|  | Non-tertiary | 3 | 378 | 97 | 19.2% (2.6-44.9) | 96.4 |  | -0.113 (-0.542 to 0.317) | 0.607 |  |
| Ciprofloxacin | Tertiary | 29 | 3488 | 807 | 22.7% (16.3-29.8) | 95.2 |  | Ref | Ref | <0.01 |
|  | Non-tertiary | 12 | 641 | 153 | 22.6% (13.9-32.6) | 86.2 |  | -0.001 (-0.147 to 0.144) | 0.987 |  |
| Levofloxacin | Tertiary | 27 | 3229 | 875 | 32.4% (22.0-43.7) | 97.5 |  | Ref | Ref | <0.01 |
|  | Non-tertiary | 9 | 608 | 282 | 38.1% (27.4-49.3) | 85.5 |  | 0.051 (-0.160 to 0.262) | 0.637 |  |

**Table 4. Results of the combined proportions of drug-resistant *Pseudomonas aeruginosa* at different regions**

|  | Regions | Proportions of resistant *Pseudomonas aeruginosa* | | | | |  | Univariate meta-regression | | |
| --- | --- | --- | --- | --- | --- | --- | --- | --- | --- | --- |
|  |  | Studies | *Pseudomonas aeruginosa* | Resistant *Pseudomonas aeruginosa* | Estimate | *I*^2^ |  | Coefficient (95% CI) | p value | *R*^2^ |
| Ampicillin | Eastern region | 7 | 399 | 394 | 99.6% (97.7-100.0) | 49.1 |  | Ref | Ref | 5.76 |
|  | Central region | 12 | 941 | 888 | 93.8% (87.4-98.2) | 89.6 |  | -0.156 (-0.351 to 0.039) | 0.117 |  |
|  | Western region | 3 | 141 | 121 | 90.6% (64.1-100.0) | 93.4 |  | -0.209 (-0.493 to 0.074) | 0.148 |  |
| Aztreonam | Eastern region | 13 | 869 | 256 | 29.7% (19.8-40.7) | 90.3 |  | Ref | Ref | <0.01 |
|  | Central region | 10 | 768 | 397 | 42.7% (9.4-80.2) | 99.1 |  | 0.131 (-0.156 to 0.418) | 0.372 |  |
|  | Western region | 5 | 1119 | 151 | 19.7% (9.3-32.5) | 93.8 |  | -0.115 (-0.470 to 0.241) | 0.527 |  |
| Cefazolin | Eastern region | 6 | 320 | 228 | 84.9% (45.1-100.0) | 98.3 |  | Ref | Ref | 11.47 |
|  | Central region | 8 | 782 | 709 | 92.7% (84.3-98.3) | 92.1 |  | 0.118 (-0.274 to 0.511) | 0.555 |  |
|  | Western region | 3 | 141 | 64 | 49.5% (9.0-90.4) | 96.8 |  | -0.382 (-0.898 to 0.133) | 0.146 |  |
| Cefepime | Eastern region | 15 | 1176 | 246 | 19.1% (11.1-28.5) | 92.5 |  | Ref | Ref | 6.75 |
|  | Central region | 13 | 1159 | 387 | 30.6% (15.9-47.4) | 96.9 |  | 0.129 (-0.044 to 0.301) | 0.144 |  |
|  | Western region | 7 | 1181 | 163 | 13.6% (9.3-18.5) | 67.5 |  | -0.089 (-0.297 to 0.119) | 0.402 |  |
| Cefoperazone | Eastern region | 1 | 25 | 2 | 8.6% (0.5-23.0) | - |  | Ref | Ref | 2.36 |
|  | Central region | 9 | 671 | 295 | 48.3% (33.4-63.4) | 92.3 |  | 0.456 (-0.129 to 1.040) | 0.126 |  |
|  | Western region | 2 | 228 | 75 | 45.0% (8.3-85.1) | 96.3 |  | 0.421 (-0.250 to 1.093) | 0.219 |  |
| Cefoperazone-sulbactam | Eastern region | 5 | 549 | 83 | 18.4% (7.9-31.9) | 91.5 |  | Ref | Ref | <0.01 |
|  | Central region | 10 | 864 | 144 | 16.8% (11.6-22.8) | 76.3 |  | -0.018 (-0.189 to 0.154) | 0.839 |  |
|  | Western region | 4 | 1041 | 67 | 9.4% (1.6-21.9) | 95.0 |  | -0.128 (-0.334 to 0.078) | 0.225 |  |
| Cefotaxime | Eastern region | 8 | 447 | 248 | 62.1% (37.9-83.6) | 96.1 |  | Ref | Ref | 0.37 |
|  | Central region | 6 | 588 | 229 | 48.6% (36.2-61.1) | 86.0 |  | -0.109 (-0.388 to 0.170) | 0.442 |  |
|  | Western region | 2 | 96 | 32 | 33.3% (24.0-43.2) | 0.0 |  | -0.285 (-0.692 to 0.122) | 0.170 |  |
| Ceftazidime | Eastern region | 18 | 1319 | 352 | 22.6% (15.1-31.1) | 91.1 |  | Ref | Ref | 4.11 |
|  | Central region | 17 | 1837 | 654 | 34.5% (22.9-47.2) | 96.4 |  | 0.128 (-0.032 to 0.287) | 0.116 |  |
|  | Western region | 8 | 1216 | 165 | 19.1% (8.7-32.2) | 94.5 |  | -0.043 (-0.243 to 0.157) | 0.671 |  |
| Ceftriaxone | Eastern region | 6 | 295 | 171 | 62.3% (23.4-93.9) | 97.8 |  | Ref | Ref | <0.01 |
|  | Central region | 11 | 902 | 530 | 67.7% (48.6-84.2) | 96.7 |  | 0.055 (-0.259 to 0.370) | 0.729 |  |
|  | Western region | 3 | 278 | 138 | 51.5% (25.5-77.2) | 93.4 |  | -0.105 (-0.539 to 0.330) | 0.637 |  |
| Imipenem | Eastern region | 18 | 1167 | 164 | 8.5% (4.1-14.1) | 87.9 |  | Ref | Ref | 5.36 |
|  | Central region | 23 | 2179 | 568 | 17.9% (9.4-28.2) | 96.7 |  | 0.138 (-0.001 to 0.276) | 0.051 |  |
|  | Western region | 9 | 1264 | 71 | 9.3% (3.0-18.2) | 92.9 |  | 0.016 (-0.163 to 0.195) | 0.859 |  |
| Meropenem | Eastern region | 12 | 1029 | 152 | 9.7% (3.7-17.7) | 92.2 |  | Ref | Ref | 4.27 |
|  | Central region | 12 | 986 | 188 | 16.7% (9.9-24.7) | 88.8 |  | 0.100 (-0.045 to 0.244) | 0.177 |  |
|  | Western region | 2 | 131 | 6 | 4.5% (1.3-9.0) | 0.0 |  | -0.100 (-0.369 to 0.169) | 0.465 |  |
| Piperacillin | Eastern region | 11 | 924 | 299 | 24.2% (10.6-41.0) | 96.3 |  | Ref | Ref | <0.01 |
|  | Central region | 13 | 1104 | 263 | 22.7% (16.1-30.1) | 83.2 |  | -0.020 (-0.206 to 0.165) | 0.830 |  |
|  | Western region | 4 | 934 | 194 | 20.2% (17.7-23.0) | 0.0 |  | -0.061 (-0.320 to 0.198) | 0.643 |  |
| Piperacillin-tazobactam | Eastern region | 9 | 692 | 114 | 11.5% (5.4-19.3) | 85.4 |  | Ref | Ref | 35.44 |
|  | Central region | 7 | 430 | 91 | 20.7% (16.2-25.6) | 26.5 |  | 0.115 (-0.020 to 0.249) | 0.094 |  |
|  | Western region | 6 | 1128 | 61 | 4.8% (1.6-9.3) | 78.6 |  | -0.126 (-0.264 to 0.012) | 0.074 |  |
| Amikacin | Eastern region | 14 | 1072 | 148 | 8.1% (2.8-15.5) | 91.9 |  | Ref | Ref | 7.14 |
|  | Central region | 21 | 1656 | 502 | 25.5% (12.6-40.9) | 97.7 |  | 0.229 (0.023 to 0.435) | 0.030 |  |
|  | Western region | 6 | 1162 | 121 | 13.8% (5.4-25.0) | 93.7 |  | 0.091 (-0.197 to 0.380) | 0.535 |  |
| Gentamycin | Eastern region | 13 | 1055 | 258 | 19.7% (11.6-29.3) | 91.7 |  | Ref | Ref | 17.50 |
|  | Central region | 17 | 1666 | 817 | 49.7% (35.1-64.4) | 97.0 |  | 0.316 (0.112 to 0.520) | 0.002 |  |
|  | Western region | 9 | 1264 | 298 | 29.4% (17.3-43.0) | 94.4 |  | 0.112 (-0.128 to 0.352) | 0.360 |  |
| Tobramycin | Eastern region | 8 | 669 | 146 | 17.2% (5.6-33.0) | 95.0 |  | Ref | Ref | 10.73 |
|  | Central region | 6 | 619 | 305 | 48.8% (12.0-86.4) | 99.0 |  | 0.337 (-0.008 to 0.681) | 0.055 |  |
|  | Western region | 5 | 443 | 91 | 20.5% (8.0-36.5) | 92.3 |  | 0.041 (-0.322 to 0.404) | 0.825 |  |
| Ciprofloxacin | Eastern region | 16 | 1268 | 258 | 17.4% (11.7-23.8) | 86.6 |  | Ref | Ref | 12.23 |
|  | Central region | 18 | 1688 | 557 | 31.4% (21.6-42.1) | 94.6 |  | 0.160 (0.027 to 0.294) | 0.019 |  |
|  | Western region | 7 | 1173 | 145 | 15.3% (8.0-24.2) | 89.0 |  | -0.026 (-0.202 to 0.150) | 0.774 |  |
| Levofloxacin | Eastern region | 12 | 928 | 206 | 22.2% (16.1-28.9) | 78.8 |  | Ref | Ref | 12.15 |
|  | Central region | 20 | 2016 | 844 | 43.3% (29.5-57.7) | 97.4 |  | 0.218 (0.032 to 0.405) | 0.022 |  |
|  | Western region | 4 | 893 | 107 | 21.0% (5.9-41.7) | 95.2 |  | -0.017 (-0.309 to 0.275) | 0.908 |  |


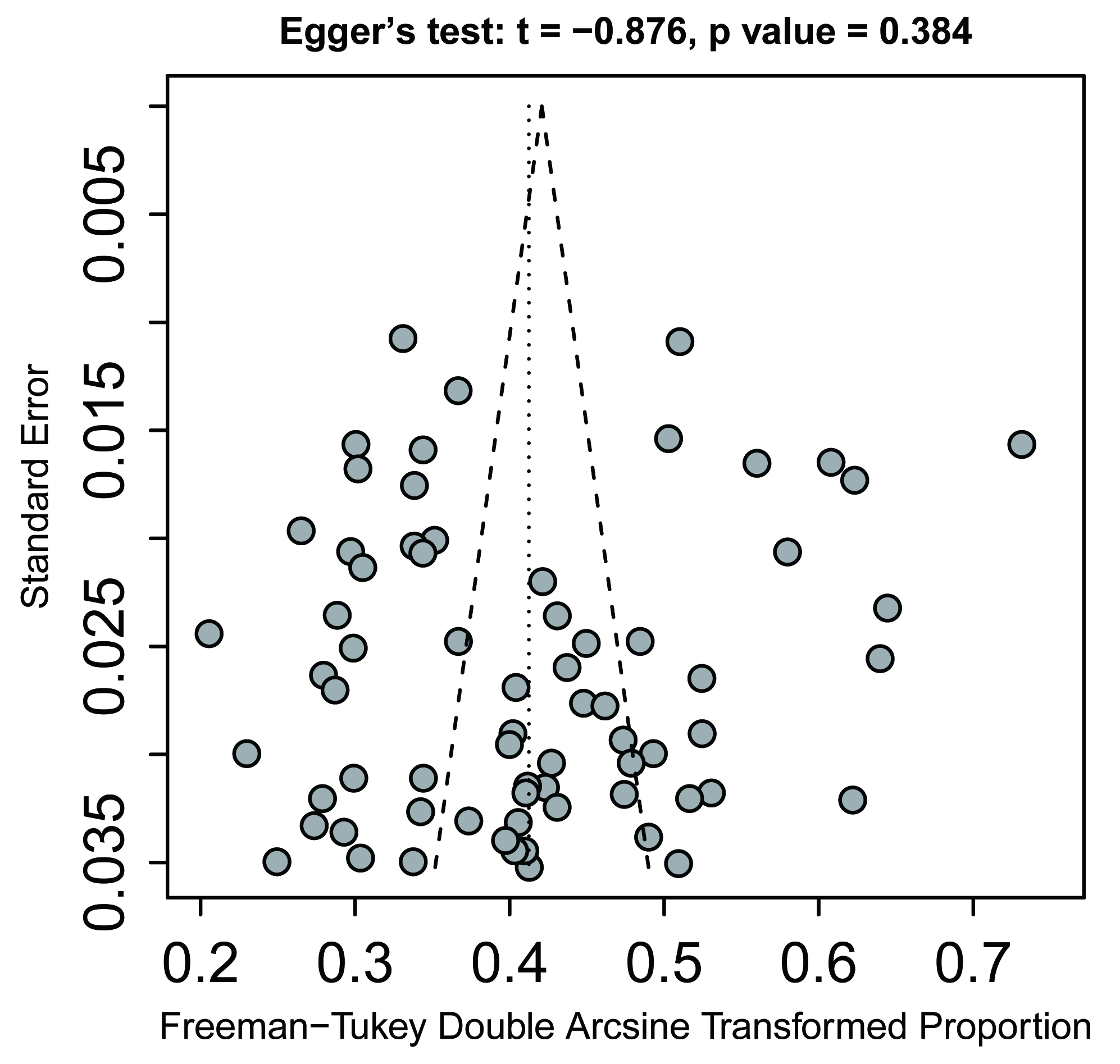


**Figure 1.** Funnel plot of the proportions of *Pseudomonas aeruginosa* among patients with SSIs.
